# Country-based report: the safety of omalizumab treatment in pregnant patients with asthma

**DOI:** 10.3906/sag-2101-341

**Published:** 2021-10-21

**Authors:** Bilun GEMİCİOĞLU, Arzu Didem YALÇIN, Yavuz HAVLUCU, Gül KARAKAYA, Levent ÖZDEMİR, Metin KEREN, Sevim BAVBEK, Dane EDİGER, İpek Kıvılcım OĞUZÜLGEN, Zeynep Ferhan ÖZŞEKER, Arzu YORGANCIOĞLU

**Affiliations:** 1 Department of Pulmonary Diseases, Cerrahpaşa Faculty of Medicine, İstanbul University-Cerrahpaşa, Istanbul Turkey; 2 Department of Internal Medicine, Division of Allergy and Immunology, Health Science University Antalya Education and Research Hospital, Antalya Turkey; 3 Department of Pulmonary Diseases, Faculty of Medicine, Celal Bayar University, Manisa Turkey; 4 Department of Pulmonary Diseases, Division of Immunology and Allergy, Faculty of Medicine, Hacettepe University, Ankara Turkey; 5 Department of Pulmonary Diseases, Dörtyol Goverment Hospital, Adana Turkey; 6 Department of Pulmonary Diseases, Süreyyapaşa Pulmonary Diseases Education and Research Hospital, Istanbul Turkey; 7 Department of Pulmonary Diseases, Division of Allergy and Clinical Immunology, Faculty of Medicine, Ankara University, Ankara Turkey; 8 Department of Pulmonary Diseases Faculty of Medicine, Uludağ University, Bursa Turkey; 9 Department of Pulmonary Diseases Faculty of Medicine, Gazi University, Ankara Turkey

**Keywords:** Exacerbation, gestation, infant, omalizumab, pregnancy, prematurity

## Abstract

**Background/aim:**

We aimed to report outcomes of pregnant patients with asthma under omalizumab treatment and their infants in our country.

**Materials and methods:**

Patients with asthma who received omalizumab for at least 6 months and at least one dose during their pregnancy were retrospectively evaluated using a questionnaire regarding their disease and therapy and the health of their infants.

**Results:**

Twenty pregnant patients and their 23 infant’s data were analyzed. The mean delivery age was 31.8 ± 7.4 years. They received omalizumab for 28.9 ± 21.8 months. Eight (36.4%) patients showed exacerbation of the disease during pregnancy. Forced expiratory volume in 1 s (FEV1) and asthma control test (ACT) scores at the starting time of omalizumab administration, first month of the pregnancy, and after delivery were 71 ± 18%, 83.4 ± 10.5%, and 80.5 ± 13% (FEV1), and 11.9 ± 4.9, 20.2 ± 2.6, and 20.4 ± 2.2 (ACT), respectively. One patient gave birth to twin infants, two patients to two infants each in different years, and 17 to one infant each. Three (13%) infants had low birth weight and five (21.7%) were born prematurely. No congenital anomalies were detected. Seven (30.4%) infants presented atopic diseases during their life.

**Conclusion:**

Omalizumab treatment during pregnancy seems to be safe for both patients and their infants.

## 1. Introduction

Asthma affects 330 million individuals worldwide and around 4 million in Turkey [1,2]. This disease has been reported in 8% of pregnant individuals [3,4]. The health condition of one-third of pregnant patients with asthma is not affected by the physiological, hormonal, and immune changes that occur during the gestation period, one-third shows improvement, and one-third shows progression and ingravescence [3,4].

There are sufficient data concerning the continuation of satisfactory asthma control during pregnancy and therefore appropriate asthma treatment for pregnant women with asthma to deliver a healthy infant [5]. In this context, many reports on omalizumab, which is used by severe allergic asthma patients during pregnancy, have been published [6,7]. The Observational Study of the Use and Safety of Xolair^®^ (omalizumab) during pregnancy (EXPECT), the largest prospective study on this subject, which has examined 228 pregnant women and 233 infants, identified a birth anomaly rate of 8.1% and preterm delivery rate of 15%. These rates are not different from the outcomes of the Quebec External Comparator Cohort (QECC) study, which involves the most extensive overall cohort of pregnant women with asthma [6]. In addition, in EXPECT vs. QECC, there was a lower rate of small for gestational age (SGA) infants of mothers receiving omalizumab (9.7% vs. 15.8%) [6]. In contrast, in the position paper of the European Academy of Allergy and Clinical Immunology (EAACI) Society regarding the administration of biological drugs for allergy during gestation, the rates of congenital anomalies and low birth weight have been reported as 4% each and that of preterm birth in singletons as 14% for omalizumab, based on the outcomes of 11 different publications [7].

Real-life data on the efficacy of omalizumab in patients with asthma have been collected and released in Turkey; however, no data related to gestation have been reported [8]. Although the case notices of all studies conducted previously across the world reported that the administration of omalizumab is safe in gestation, the number of pregnant patients in these case series was between one and four, and there was only one observational study from the United States [6,7]. In our study, we worked with the real-life data sample reported from one country: 22 pregnancies and 23 infants. Therefore, we aim to share our experience regarding the safety of omalizumab in both mothers and infants by a retrospective review of the relevant data from Turkey.

## 2. Materials and methods

This retrospective real-life case study was approved by the Institutional Ethic Committee (06.08.2019 date 83045809-604.01.02-123451 number); Helsinki Declaration was signed by all coauthors. Informed consent was obtained from each patient.

The centers participating in the Turkish omalizumab data survey were called for participation in this study [8]. Nine centers participated in the study as they had pregnant patients under omalizumab therapy with born infants. This study was conducted in Turkey from August 15, 2019 to October 31, 2019. A structured questionnaire was prepared by the authors to collect information regarding demographic and clinical parameters of the mothers as follows; age, living area, smoking status, body mass index, occupation, education level, prick test results and total IgE levels at the beginning of omalizumab administration; spirometry, asthma control test (ACT), and whole blood count results at the beginning of omalizumab administration, beginning of the pregnancy, and after the delivery of the infant; exacerbation rate and exacerbation trimester time during pregnancy and last year before pregnancy; treatment-related parameters (adherence); infant demographic parameters and health during the whole breastfeeding period, until the end of the study. Patients with asthma who received omalizumab for at least 6 months and at least one dose during pregnancy and had a live birth were retrospectively evaluated using a questionnaire. The data not present in the file of the patient were collected via the questionnaire prepared by the authors when the patients came to the relevant clinics for their omalizumab injections after having their informed consent. The outcome or the safety of omalizumab was attributed as the control status and the exacerbation rate of the pregnant patient, the APGAR score, the prematurity, the birth weight, the rate of malformation, and the allergic diseases of the infants.

The data of the patients are presented as exact numbers and percentages. If numerical variables are normally distributed, they are presented as mean ± standard deviation. Nonnormally distributed data are presented as median (min-max). The evaluation of spirometry data, ACT scores, and whole blood count data at different time points were compared using the Friedman variance analysis and the Wilcoxon signed rank test. Pearson’s correlation test was used when two variables were continuous. All data were evaluated within a 95% confidence interval with a significance level of p < 0.05. All statistical analyses were performed using SPSS Version 20.0 (IBM Corp. 2012).

## 3. Results

The data correspond to 20 pregnant patients with asthma (22 singleton pregnancies, 1 twin pregnancy) and their 23 infants. One patient gave birth to twin infants, two patients to two infants each in different years, and 17 to one infant each when they were on omalizumab. 

### 3.1. Maternal demographics and clinical characteristics

Four patients (18%) were over 34 years old. The majority of the patients lived in urban areas (95.5%), and 59% were housewives. The mean asthma duration before treatment with omalizumab was 12.2 ± 8.1 years (range, 2–40 years). All patients were allergic to house dust mite (Dermatofagoides pteronissymus). Five of them had also mold allergy (alternaria) and 2 of them also had cockroach positive skin prick test. Their total serum IgE level was 242.1 ± 198.8 IU in the beginning of omalizumab treatment. Exacerbation rate (requiring oral corticosteroid treatment for 3 or more days) last year before pregnancy was 40%. Only eight (36.4%) of the patients showed exacerbation (requiring oral corticosteroid treatment for 3 or more days) during pregnancy and half of the exacerbation were in the third trimester. All of them used inhaled corticosteroids (ICS) and long-acting beta-agonists (LABA) with high adherence level in 86.4% of the pregnancies. Demographic and clinical data of the patients are presented in Table 1.

**Table 1:** Maternal demographics and clinical and therapeutic characteristics of 22 pregnancies.

Age (years)	
Mean ± standard deviation (SD)	31.8 ± 7.4
N, % <34 y	4 (18%)
Living area (N and %)	
Urban	21 (95.5%)
Rural	1 (4.5%)
Education (N and %)	
Illiterate	2 (9%)
Primary school	6 (27.3%)
High school	7 (31.8%)
University	7 (31.8%)
Occupation (N and %)	
Housewife	13 (59%)
Officer	7 (31.8%)
Self-employed	2 (9.1%)
Asthma history before omalizumab (years)	
Median (range: min–max)	10 (2–40)
Smoking history (N and %)	1 patient ex-smoker (5 packs-year), 4.3%
Body mass index (BMI) (kg/m2	
Median (range: min–max)	24 (20–37)
BMI > 30 (obesity) ( N, %)	5 (22.7%)
Comorbidities (N and %)	
Allergic rhinitis	16 (72%)
Exacerbation rate before pregnancy (last year, with oral corticosteroids) (N and %)	9 (40%)
Exacerbation rate during pregnancy (with oral corticosteroids) (N and %)	8 (36.4%)
1 exacerbation case (N and %)	3 (13.6%)
2 exacerbation cases (N and %)	5 (22.7%)
Exacerbation time during pregnancy	
First trimester (N and %)	3 (37.5%) of 8
Second trimester (N and %)	1 (12.5%) of 8
Third trimester (N and %)	4 (50%) of 8
Medications other than omalizumab (N and %)	
Inhaled corticosteroid (ICS)	2 (9.1%)
ICS + long acting beta agonist (LABA)	22 (100%)
Leukotrien receptor antagonists (LTRA)	15 (68.2%)
Tiotropium	2 (9.1%)
Histamine1 receptor antagonists*	6 (27.3%)
Nasal corticosteroids	9 (40.9%)
Adherence to medications other than omalizumab (N and %)	
High adherence**	19 (86.4%)
Medium adherence**	2 (9.1%)
Low adherence**	1 (4.5%)
Nonadherence**	0

### 2.2. Maternal spirometry, ACT, and whole blood count results

Spirometry parameters, ACT, and whole blood count results at the starting time of omalizumab administration, during pregnancy, and after delivery are presented in Table 2. ACT score at the starting time of omalizumab administration was statistically lower than the beginning of pregnancy and after delivery. There were no considerable differences between other results at the beginning of pregnancy and after delivery. No correlation was found between forced expiratory volume in 1 s (FEV1) and the gestational week at the birth of the infant (p > 0.05).

**Table 2:** Spirometry parameters, asthma control test (ACT) scores, and whole blood count results (mean ± standard deviation (SD)).

	Starting time of omalizumab	Beginning ofpregnancy	After pregnancy(1–3 months)	p
Spirometry	N: 22 (100%)	N: 19 (86.4%)	N: 21 (95.5%)	
FVC (L)	3.05 ± 0.55	3.61 ± 0.55	3.21 ± 0.49	>0.05
FVC (%)	83.6 ± 18.8	92.3 ± 12.2	89.9 ± 10.0	>0.05
FEV1 (L)	2.39 ± 0.80	3.00 ± 0.95	2.62 ± 0.78	>0.05
FEV1 (%)	71.0 ± 18.2	83.4 ± 10.5	80.5 ± 13.0	>0.05
FEV1/FVC	76.8 ± 20.4	87.2 ± 21.5	89.9 ± 10.0	>0.05
ACT (N: 22, 100%)	11.95 ± 4.95	20.16 ± 2.64	20.40 ± 2.18	<0.05
Whole blood count	N: 22 (100%)	N: 19 (86.4%)	N: 21 (95.5%)	
Hemoglobin(g/dL)	12.9 ± 1.0	12.3 ± 1.2	12.1 ± 1.5	>0.05
Hematocrite (%)	37.6 ± 3.7	35.9 ± 3.9	34.4 ± 3.9	>0.05
MPV	9.5 ± 1.8	9.4 ± 1.8	9.6 ± 1.7	>0.05
Trombocytes/mm3	271 545 ± 63 120	273 263 ± 63 625	262 571 ± 59 815	>0.05
Eosinophils/mm3	447.8 ± 297.1	356.1 ± 246.9	226.7 ± 198.7	>0.05
Eosinophils (%)	4.58 ± 2.74	2.92 ± 1.98	1.81 ± 1.79	>0.05

### 2.3. Omalizumab exposure results

Omalizumab exposure level, interval, and duration are presented in Table 3. The patients had asthma for 12.2 ± 8.1 years and they received omalizumab for 28.9 ± 22.3 months. The twins received only one dose of omalizumab, one infant received the drug in the first trimester, four infants in the second and third trimesters, and 16 infants throughout the pregnancy and breastfeeding periods (minimum 3 months, maximum 28 months with a median 13 months).

**Table 3:** Omalizumab exposure, pregnancy, and infants outcomes.

Omalizumab exposure	
Time before pregnancy (months) (mean ± SD)	28.9 ± 22.3
Dose (mg)	
150 (N and %)	4 (18.2%)
225 (N and %)	2 (9.1%)
300 (N and %)	11 (50%)
375 (N and %)	2 (9.1%)
Other (N and %)	3 (13.6%)
Dose interval	
Every 2 weeks (N and %)	5 (22.7%)
Every 4 weeks (N and %)	17 (77.3%)
Exposure time	
1st trimester (N and %)	3 (13.6%)
2nd trimester + 3rd trimester (N and %)	4 (18.2%)
All trimesters and breastfeeding period (N and %)	16 (69.6%)
Pregnancy and infant outcomes	
Birth weight (mg) (mean ± SD)	
All infants	3055.8 ± 563.3
Singletons	3109.1 ± 540.3
Twins	2062.5 ± 12.5
Low birth weight* (N and %)	
All infants	3 (13.04%)
Singletons	1 (4.76%)
Twins	2 (100%)
Birth height (cm) (mean ± SD)	48.2 ± 3.7
Gestational age (weeks) (mean ± SD)	37.3 ± 2.2
Premature birth** (N and %)	5 (21.7%)
Sex: male/female (N and %)	9/14 (39.1/60.9%)
APGAR Score	
Median (range: min–max)	9 (5–10)
Low APGAR score*** (N and %)	1 (4.76%)
Infants with any allergic diseases (N and %)	6 (26.09%)
Actual age of infants (weeks) (mean ± SD) (range)	31.2 ± 28.9 (range: 7–120)

*Low birth weight was defined as <2.5 kg.

### 2.4. Pregnancy and infant outcomes

Ten patients were delivering their first infant. Seventeen (73.9%) patients underwent a cesarean section. No labor complications were noted. Pregnancy and infant outcomes are given in Table 3. The mean maternal age at delivery was 31.8 ± 7.2 years. No congenital anomalies were detected. Seven infants had allergic diseases during their lives. The median APGAR score for newborn infants was 9 (maximum 10, minimum 5). Only one newborn infant had an APGAR score below 8 (5). The mean birth weight was 3055.8 ± 563.3 g. Premature births were seen in five patients (21.7%).

## 3. Discussion

In our study of 22 pregnancies and 23 infants, asthma exacerbation was seen only in 36.4% of the pregnancies. FEV1 levels, ACT scores, and eosinophilia at the start of pregnancy were not statistically significantly different than those after pregnancy (p > 0.05). The patients received omalizumab for 28.9 ± 22.3 months before pregnancy. The twins received only one dose of omalizumab, one infant received it in the first trimester (two doses), four infants in the second and third trimesters, and 16 infants throughout the pregnancy and breastfeeding periods. No malformations were detected in the newborns and only one newborn had an APGAR index below 8. Nevertheless, premature birth was seen in five infants (21.7%) and seven infants had allergic diseases during their life.

Maternal age is an important risk factor for congenital anomalies. The median age of our patients was 31 years (range, 25–47 years). It was equal to that in the EXPECT cohort and higher than that in the QECC cohort (31 and 27.7, respectively) [6]. Furthermore, the percentage of subjects younger than 35 years was 82% in our study but 74.8% in the EXPECT cohort and 85.7% in the QECC cohort [6]. However, the infants in our study did not present any anomalies.

The majority of our patients were from urban areas (95%), 59% were housewives, and 63.6% were high school or university graduates. Compared with other pregnant groups studied in our country, our pregnant patients with asthma were more educated, had different occupations, and were less commonly from rural areas [9]. Coming from urban areas means more exposure to traffic pollution, which may lead to preterm birth [10].

Only one patient was an ex-smoker. The major comorbidity was allergic rhinitis (72%), and only five patients (22.5%) were obese. In contrast, in the EXPECT study, the rate of obese patients was 46.7% [6]. Furthermore, our patients did not have comorbidities such as diabetes, which might have increased the risk of certain lung diseases in infants [11]. Maternal allergy was not present in all infants. Only seven infants had allergic diseases at time of the study.

Asthma duration before omalizumab administration had a range of 2 to 40 years, and all the patients had been using ICS and LABA combinations before and during their pregnancy. Other add-on drugs used during pregnancy were also nonteratogenic; patients had used similar drugs in EXPECT and QECC studies [6]. Moreover, whereas Yilmaz et al. pointed out nonadherence to treatment in pregnant asthma patients, we did not encounter it in our patients [9]. High adherence to medications was found in our study.

Exacerbation was observed in eight (36.4%) of our patients. This rate is lower than that before pregnancy. In a study conducted by Schatz et al., with 1739 pregnant patients with asthma, 30% of the patients who had previously been identified as having mild asthma had progressed towards moderate to severe asthma, but in subjects with severe asthma, only 23% had progressed towards moderate and mild asthma [12]. Furthermore, Schatz et al. showed that 13% of mild, 16% of moderate, and 52% of severe asthma cases presented at least one exacerbation case [12]. This suggests that there is a correlation between asthma severity and increased risk of exacerbation. The exacerbation was reported to occur mostly between weeks 17 and 24; in other words, mostly in the second trimester of gestation [3]. In conclusion, even though pregnant women with severe asthma are considered to be at higher risk of an attack, the ones with mild asthma are also at risk.

Furthermore, it has been demonstrated that inflammation is associated with a risk of lack of asthma control, which is due to failure to administer ICS, decrease in treatment adherence, or obesity [13]. Uncontrolled maternal asthma results in poor outcomes in infants [14]. Spirometry parameters, ACT scores, and whole blood count results at the beginning of pregnancy and after delivery showed nonconsiderable differences in our patients. However, one-third of the exacerbation cases resulted from oral steroid use. In contrast, an exacerbation rate of up to 45% in pregnant patients with asthma has been reported [4]. However, their exacerbation rate, ACT score, and FEV1, as well as blood test results at the beginning of pregnancy were not different from the ones after pregnancy (p > 0.05); the decrease in eosinophil count after pregnancy was also not significant (p > 0.05). Similarly, Fazel et al. found nonconsiderable differences in eosinophil levels between controlled and uncontrolled pregnant patients with asthma and healthy pregnant women [15]. Meanwhile, Palmsten et al. used a modified ACT (p-ACT) [16]. They reported that lower p-ACT scores were associated with previous exacerbation, and were not associated with future exacerbation during pregnancy. Furthermore, De Araujo et al. evaluated the importance of ACT in pregnancy and pointed out that physicians did not require spirometry to assess the level of asthma control and that ACT can be used in the primary care of expectant mothers with asthma [17].

The gestational median age was 37.3 years in our patients, and there was a higher rate of cesarean section (73.9%) in our study patients. Furthermore, five infants (21.7%) had premature birth and three (13% of all infants) had low birth weight. Compared to the EXPECT study, the low birth weight rate was similar, but the premature birth rate was higher in our study [6]. As mentioned in EXPECT study among women who took any oral corticosteroid at any time during pregnancy, the prevalence of premature birth was 32.7% in their subcohort [6]. All of our five patients with premature birth used oral steroids for their exacerbation. Additionally, only one infant had a low APGAR score, and we found that the seven infants with allergic diseases demonstrated genetic predisposition, and omalizumab did not have an effect in this situation. Meanwhile, no considerable differences between mothers with and without asthma regarding the duration of gestation, birth weight, low APGAR scores, or neonatal respiratory difficulties were found in the study by Fazel et al. [15]. Furthermore, none of our patients had infants with congenital anomalies; however, this may be due to our small sample size. In fact, in 2008, Blais and Forget reported that the malformation probability in 4300 pregnant women with asthma was increased [18]. It has been reported that this is particularly correlated with exacerbation in the first trimester (odds ratio 1.48, 95% CI 1.04–2.09) [18]. The most notable outcomes in this regard have been suggested by a metaanalysis consisting of 40 studies and 1,637,180 individuals conducted by Murphy et al. in 2011 [19]. In this study, it was observed that the risk of low birth weight infants was increased in pregnant women with asthma [relative risk (RR) 1.46, 95% confidence interval (CI) 1.22–1.75], there was a slight decrease in intrauterine growth (IUGR) (RR 1.22, 95% CI 1.14–1.31), preterm delivery was increased (RR 1.41, 95% CI 1.22–1.61), and preeclampsia risk was also slightly higher (RR 1.54, 95% CI 1.32–1.81) [19]. It has been reported that IUGR and preterm delivery are due to maternal hypoxemia and altered placental function in asthma [20]. We did not find any correlation between FEV1 and prematurity. In the study conducted by Murphy et al., it was suggested that preterm delivery and preeclampsia decreased upon ensuring control through proper asthma treatment [19]. Thus, it can be concluded that such complications occur due to the lack of proper treatment.

There are some limitations to our study. As the study was retrospective, we could not obtain all the data for the whole duration of the pregnancies. Moreover, other patients on omalizumab during pregnancy may exist in our country; we presented only those cases for which the physicians agreed to participate in the study.

In conclusion, about one-third of our patients with severe asthma on omalizumab had exacerbation during pregnancy. However, the spirometry, ACT scores, and blood count results after the delivery of the infants were not considerably different from those before pregnancy. Two-thirds of our patients received omalizumab throughout the gestation and breastfeeding periods, and premature birth was seen in one-fifth of the patients. Overall, omalizumab treatment during pregnancy seems to be safe for both patients and their infants.

## Funding

This study has received no financial support.

## Informed consent

The study was approved by the Institutional Ethic Committee of Cerrahpaşa Medical Faculty (06.08.2019 date 83045809-604.01.02-123451 number); Helsinki Declaration was signed by all coauthors. Informed consent was obtained from each patient.
